# Efectividad de fotopolimerización usando lámparas led: una revisión

**DOI:** 10.21142/2523-2754-1003-2022-120

**Published:** 2022-09-28

**Authors:** Alexandra Jacqueline Aquino Valverde, Gina del Pilar Aguilar Vargas, José Miguel Díaz Fernández, Piero Aryed Leiva Ramírez, Daleska Adriana Quintanilla Labajos, Katherine Joselyn Atoche Socola, Marcia Vidalón Pinto

**Affiliations:** 1 Facultad de Odontologia, Universidad Cientifica del Sur. Lima-Peru. aaquinovalverde@gmail.com, ginadelpilar2001@gmail.com, josedf7@gmail.com, pieroleiva2020@gmail.com, dquintanillalabajos@gmail.com, mvidalon@cientifica.edu.pe Universidad Científica del Sur Facultad de Odontologia Universidad Cientifica del Sur Lima Peru aaquinovalverde@gmail.com ginadelpilar2001@gmail.com josedf7@gmail.com pieroleiva2020@gmail.com dquintanillalabajos@gmail.com mvidalon@cientifica.edu.pe; 2 Division de Rehabilitacion Oral, Facultad de Odontologia, Universidad Cientifica del Sur. Lima-Peru. katoche@cientifica.edu.pe Universidad Científica del Perú Division de Rehabilitacion Oral Facultad de Odontologia Universidad Cientifica del Sur Lima Peru katoche@cientifica.edu.pe

**Keywords:** polimerización, lámparas de polimerización dental led, resinas dentales, fotoiniciadores, polymerization, LED dental curing lights, dental resins, photoinitiators

## Abstract

**Introducción:**

Las lámparas led tienen una tecnología nueva de fotocurado, y pueden ser *monowave* o *polywave*, lo cual les permite llegar a más iniciadores como canforquinona, lucerina TPO y PPD, que presentan una gran variedad de ventajas y desventajas. Estas lámparas han evolucionado con el tiempo, al igual que tienen diferentes ergonomías, longevidades, sistemas y estándares de calidad.

**Objetivo:**

El objetivo de esta revisión de literatura es actualizar al clínico en el uso adecuado de las diferentes lámparas led y cómo estas influyen en la eficiencia de la fotopolimerización de la resina.

**Material y métodos:**

Se ha realizado una amplia investigación en la literatura existente sobre este tema. Desde el inicio de esta información hasta el 18 de abril de 2022, la búsqueda bibliográfica realizada incluye 86 artículos publicados en la base de datos de Medline a través de PubMed, LILACS, Science Direct y SciELO, y no existe restricción de idioma. Incluyen investigación observacional, descriptiva, longitudinal, revisiones sistemáticas, libros y editoriales.

**Resultados:**

Los efectos de la fotopolimerización de las lámparas led *polywave* y *monowave* presentan diferencias significativas en la resistencia a la compresión de las resina fotopolimerizadas, con lámpara led de onda única y polivalente donde los tipos de luz y lámpara influyen directamente en la resistencia a la compresión de las resinas compuestas.

**Conclusión:**

El tipo de luz y lámpara afecta directamente la efectividad de la fotopolimerización de la resina compuesta, por lo que se concluye que las lámparas led con tecnología de onda única (*monowave*) producen una mayor profundidad de fotopolimerización que las de tecnología de onda múltiple (*polywave*).

## Abstract (en)

**Introduction:**

LED lamps have a new light-curing technology which can be monowave or polywave, which allows it to reach more initiators such as camphorquinone, Lucirin TPO and Propanodione, which have a wide variety of advantages and disadvantages. These lamps have evolved over time, as have different ergonomics, longevity, systems and quality standards.

**Objective:**

The objective of this literature review is to improve the clinician on the proper use of different LED lamps and how they influence the efficiency of resin photopolymerization.

**Material and methods:**

Extensive research has been carried out in the existing literature on this topic. From the beginning of this information until April 18, 2022, the bibliographic search carried out includes 86 articles published in the Medline database through PubMed, LILACS, Science Direct and SciELO, and there is no language restriction.

**Results:**

The photopolymerization effects of Polywave and Monowave LED lamps present significant differences between the compressive strength of the light-cured resin, with single-wave and polyvalent LED lamps where the types of light and lamp directly influence the compressive strength of the resin. composite resins.

**Conclusion:**

The type of light and lamp directly affects the efficiency of the photopolymerization of the composite resin, so it is concluded that LED lamps with single wave technology (Monowave) produce a greater depth of photopolymerization than those with multiple wave technology (Polywave).

## INTRODUCCIÓN

El inicio de una nueva era en los años 70 trajo consigo la llegada de las primeras resinas compuestas; asimismo, de las lámparas de fotopolimerización, las cuales han ido evolucionando constantemente a lo largo de los años ^1^. No fue sino hasta 1995 que Mill ^2^ publicó la primera mención del uso de luz led como lámpara de fotopolimerización en el campo dental. Lo cierto es que la eficacia de las resinas compuestas dependerá de la correcta fotopolimerización, lo que aumentará su vida media y maximizará el rendimiento de su matriz para reducir su grado de contracción. Las lámparas led tienen una tecnología nueva de fotocurado y pueden ser *monowave* o *polywave*, lo cual les permite llegar a más iniciadores como canforquinona, lucerina TPO y PPD, que presentan una gran variedad de ventajas y desventajas. Estas lámparas han evolucionado con el tiempo, al igual que tienen diferentes ergonomías, longevidades, sistemas y estándares de calidad ^3^.

En el proceso de fotopolimerización, es de gran relevancia tener en cuenta los tipos e intensidades de luz que existen. Zorzin ^4^ menciona que el tiempo de exposición adecuado para lograr propiedades óptimas es de 30 segundos, considerando las ventajas de una lámpara led, ya que consume menos energía, no requiere ventiladores de refrigeración y tiene una vida útil prolongada sin perder su intensidad de luz en el proceso. La fotopolimerización comienza rápidamente después de la irradiación de luz y continúa después de que finaliza su emisión, e incluso se describe que el proceso de fotopolimerización continúa 24 horas después de la irradiación ^5^. Por lo tanto, se distinguen dos fases: una lumínica y una oscura. Algunos autores, como Davidson y Feilzer ^6^, mencionan que la fase lumínica fotopolimerizada pasa por tres fases importantes: la fase pregel, el punto gel y la fase posgel. La primera inicia la fotopolimerización; la segunda ocurre durante el proceso mismo de fotopolimerización, en el cual se formarán macromoléculas para pasar a un estado sólido; y, por último, la tercera, en cual la resina se encontrará en un estado elástico rígido y se llevará a cabo su contracción ^7^.

Posteriormente, la fase oscura de la fotopolimerización es aquella en la cual la resina aún continúa fotopolimerizando y genera un aumento en la conversión. Esta fase comienza tan pronto como la luz se desvanece y la iluminación cerca de la resina se retira, y dura al menos 24 horas después de que el estímulo de la luz se desvanece, incluso en la oscuridad total. Es importante señalar que esta etapa solo ocurre después de que la resina ha sido expuesta a la fuente de luz, por lo que ya ha reaccionado a la luz anterior ^8^.

Existen diversos materiales dentales: los autopolimerizables, que dependen de una reacción química, y los fotopolimerizables, que dependen de luz para su conversión de monómero a polímero. Por consiguiente, se ha determinado que la fuente de luz, la interposición de la resina y el tiempo de exposición de luz pueden afectar la profundidad de la fotopolimerización ^9^.

Actualmente, existe una gran variedad de resinas nanohíbridas, diseñadas para imitar la apariencia de los dientes y cumplir las expectativas estéticas de los pacientes. Esto se relaciona directamente con la coincidencia y estabilidad de los colores. Además, uno de los objetivos de la odontología estética es obtener una restauración que mantenga el color durante el tiempo, de manera que se evite el gasto innecesario al paciente por la sustitución de la restauración dental ^10^. Además, las resinas nanohíbridas, al estar compuestas por micropartículas, producirán menor contracción cuando se fotopolimerizan, lo que genera menor tensión de contracción en las restauraciones ^11^. Asimismo, el factor C comprende las superficies adheridas y las no adheridas de una cavidad preparada, lo que tiene una estrecha relación con los resultados de la fuerza de contracción de la resina, ya que esto permite colocar incrementos de pequeñas cantidades de resina, lo cual favorece la calidad y el éxito de la restauración ^12^. Cabe resaltar que la fotopolimerización inadecuada provoca situaciones clínicas indeseables, como decoloraciones amarillentas, irritaciones pulpares, sensibilidad posoperatoria o fallas de restauraciones ^13^.

Por ello, la presente revisión busca actualizar al clínico en el uso adecuado de las diferentes lámparas led y cómo estas influyen en la eficiencia de la fotopolimerización de la resina.

## MATERIALES Y MÉTODOS

Se ha realizado una amplia investigación en la literatura existente sobre este tema. Desde el inicio de esta información hasta el 18 de abril de 2022, la búsqueda bibliográfica realizada incluye 39 artículos publicados en la base de datos de Medline a través de PubMed, LILACS, Science Direct y SciELO, y no existe restricción de idioma. Las palabras clave utilizadas son: *polymerization*, *LED dental curing lights*, *dental resins y photoinitiators*.

Incluye investigación observacional, descriptiva, longitudinal, revisiones sistemáticas, libros y editoriales. Para ser incluidos, los estudios deben describir la efectividad de la fotopolimerización haciendo uso de las lámparas led.

## RESULTADOS

### Evolución de las lámparas dentales

Una de las primeras fuentes lumínicas fue la lámpara de luz halógena, la cual presentaba una longitud de onda de entre 400 y 550 nm y una intensidad de 400 a 800 mw/cm^2 (^^14^, aproximadamente, lo que permite abarcar la fenilpropanodiona (PPD) y también la canforquinona. Entre las desventajas de esta lámpara está que tiene una vida limitada de entre 40 a 100 horas, ya que presenta estructuras que se deterioran rápidamente debido a la alta temperatura que emite y sin necesidad de haber pasado mucho tiempo ^16^. La lámpara de arco de plasma tuvo el principal objetivo de reducir el tiempo de exposición y realizar una fotopolimerización más profunda ^17^, pues posee un rango de onda de entre 460 y 480 nm, y una intensidad de 2400 mw/cm^2^, parámetros similares a los de la canforquinona ^18^. Para evitar el calor excesivo generado por esta lámpara, se recomienda utilizarla en obturaciones con incrementos de 2 mm en un intervalo de 3 a 5 segundos. De igual manera, entre las desventajas que presenta esta lámpara se encuentran el ruido que emite, su alto costo y su tamaño, que la hace poco práctica para transportar ^19^. Las lámparas de tipo argón poseen una intensidad de 800 mw/cm^2^ y un rango de onda de 476,5 a 480 nm, compatibles con los materiales que presentan como fotoiniciador a la canforoquinona ^20^, pero son poco utilizadas porque emiten mucho calor y generan contracción de fotopolimerización elevada en los materiales ^21^. A finales de los 90, aparecieron las primeras lámparas de diodo emisor de luz (lámparas led), que comenzaron a utilizarse en la odontología como lámparas de fotopolimerización. Estas no tienen incorporado un filtro en su conformación ni fibra óptica, y brindan una mayor fuente luminosa que otras fuentes lumínicas ^22^. La lámpara LED de primera generación surgió en 1999, con un rango de onda de 465 a 470 nm, presentaba baja intensidad de luz (100-400 nW/cm)^25^ que nos permite abarcar a la canforoquinona y la fenilpropanodiona ^26^. Las lámparas LED de primera generación, en comparación con las lámparas de luz halógena, emitían una radiación muy intensa, por lo que se les incorporó disipadores de calor y nuevos chips más planos y pequeños, los cuales ayudaron a optimizar la salida de luz y maximizar la emisión de calor ^27^. En 2002, las lámparas de segunda generación se lanzaron al mercado con una intensidad de 500 a 1400 mW/cm y un rango de onda de 420 a 490 nm. Esta lámina permite obtener una profundidad similar a las lámparas de luz halógena, pero con un menor tiempo de exposición, por lo que son capaces de fotopolimerizar de manera exitosa materiales hechos con canforquinona; sin embargo, la fotopolimerización en los materiales que contenían fenilpropanodiona no fue tan exitosa ^25^. Las lámparas de tercera generación o *polywave*, disponibles en el mercado desde 2004, son capaces de alcanzar una intensidad de 3200 mW/cm ^28^. La ubicación de los chips de luz y los led permite una mayor longitud de onda, lo cual permite fotopolimerizar todos los materiales restauradores independientemente del fotoiniciador que presenten, sin alterar su composición. Al utilizar baterías de litio, permiten un mayor tiempo de uso y rendimiento en la práctica odontológica ^29^.

### Fotoiniciadores

Existen dos tipos de sistemas de fotoiniciadores presentes en las resinas compuestas: fotoiniciadores Norrish tipo I, que generan radicales libres al disociar el fotoiniciador generando dos o más radicales libres, y fotoiniciadores Norrish tipo II, como canforquinona, con coiniciadores que generan radicales libres capaces de iniciar la fotopolimerización ^30^. Los compuestos derivados de los óxidos de fosfina, como el óxido de monoacilfosfina (Lucerin), la fenilpropanodiona (PPD) y el benzoilgermanio (Ivocerin), se han utilizado como sustitutos de la canforquinona en resinas compuestas. Sin embargo, estos fotoiniciadores alternativos tienen dispositivos de fotocurado que emiten luz violeta reducida para reparar estos fotoiniciadores ^31^.

Por lo tanto, por razones estéticas, se introdujeron nuevos sistemas iniciadores en las resinas compuestas, ya que tienen un color más claro que la canforoquinona y mejoran la estabilidad del color, lo que permite seleccionar el tono correcto de la resina que se ajuste a los dientes naturales de una forma más fácil ^32^. Por otro lado, los fotoiniciadores Norrish de tipo I a partir de óxidos de fosfina no requieren un coiniciador para iniciar la fotopolimerización, lo que reduce en gran medida el efecto de amarillamiento a largo plazo causado por la oxidación de las aminas terciarias. Asimismo, se excitan con la luz de longitud de onda específica y, como resultado, se dividen en radicales libres que provocan la fotopolimerización de los monómeros ^33^.

El fotoiniciador canforquinona es el más utilizado en resinas fotocurables y tiene un pico de absorción en el rango visible del espectro azul con una longitud de onda central de 468 a 470 nm ^34^. La canforquinona es amarilla bajo la luz blanca y solo una parte del contenido se usa realmente en la resina fotopolimerizable, razón por la cual las restauraciones tienden a ser amarillentas ^35^. Cuando se expone a una luz de longitud adecuada, la canforoquinona absorbe un fotón para generar un estado excitado que forma un complejo con una amina terciaria, lo que facilita la transferencia secuencial de electrones y protones, y da como resultados radicales que inician la fotopolimerización ^36^.

La fenilpropanodiona es un fotoiniciador de color claro o translúcido con un espectro de absorción en el rango de 390 a 450 nm, y un pico máximo de absorción a 410 nm. Este fotoiniciador, a menudo, se combina con la canforquinona para producir un efecto sinérgico, lo que da como resultado una mejor fotopolimerización de la resina y reduce el amarillamiento residual de las restauraciones ^37^.

El fotoiniciador Ivocerin fue desarrollado por Ivoclar para su composite Bulk-fill, y constituye el 0,2% de la composición de la resina. Similar a la canforquinona amarilla, cuando se combina con ella se obtiene una fotopolimerización más completa ^38^. Tiene una actividad de fotoactivación más alta que la canforquinona y absorbe la luz visible en el espectro violeta a 380-420 nm; por lo tanto, se superpone principalmente al pico de emisión del espectro violeta a 410 nm. Además, se puede utilizar sin la acción de aminas terciarias como coiniciadores para generar radicales libres. Así, se puede considerar beneficioso agregar una gran cantidad de material en un solo incremento ^39^.

### Fotopolimerización

La resina compuesta se fotocura mediante fotocuración radial, los fotones son absorbidos por el fotoiniciador, que convierte la energía de la luz en energía química mediante la formación de radicales libres que rompen el doble enlace carbono-carbono en el monómero, lo que permite que la reacción forme un único polímero unido covalentemente. Sin embargo, la fotopolimerización es la conversión de monómeros en polímeros iniciada por fotoiniciadores tras la exposición a la luz de longitudes de onda compatibles ^40^.

Esta reacción química se debe a la transformación de los radicales libres que la inician, cuando dos o más monómeros diferentes se fotopolimerizan juntos, es decir, cuando los monómeros se combinan químicamente para formar macromoléculas, cientos de las cuales forman un polímero ^41^.

La fotopolimerización consta de tres etapas: la iniciación, la propagación y la terminación. En la primera, se activa la luz visible, el fotoiniciador de la resina compuesta genera radicales libres bajo la irradiación de una longitud de onda específica, y los radicales libres se difunden en el medio de la resina, en busca de áreas con un gran número de electrones, formando así enlaces dobles de carbono-carbono de monómeros a base de metacrilato ^42^. En la segunda etapa, los radicales libres de los monómeros de metacrilato se combinan con monómeros adyacentes para extender la longitud de la cadena, puesto que forman un radical libre de monómero que reaccionará continuamente con otras moléculas de monómero, lo que aumenta el crecimiento del polímero a medida que continúa la fotopolimerización en cadena. Estas cadenas de polímeros se van a crear en diferentes formas estructurales ^43^. En la última, a medida que avanza la reacción, la concentración de monómeros disponibles disminuye y la creciente cadena de radicales libres se vuelve cada vez más difícil de difundir a través de la matriz de resina. Por lo tanto, el crecimiento de las cadenas se detiene debido a la combinación de electrones libres de las dos cadenas en crecimiento y se forman enlaces intermoleculares, con lo que finaliza el proceso de fotopolimerización ^44^.

### Polímeros y luz

Los polímeros sintéticos se encuentran entre los materiales más importantes en la sociedad moderna; la luz presenta una gran oportunidad para la ciencia de los polímeros. La luz se puede utilizar para iniciar y conducir reacciones de fotopolimerización, mientras que los materiales macromoleculares se han diseñado para aprovechar la energía de la luz, responder a sus estímulos o, incluso, controlar su propagación ^45^. La capacidad de fotopolimerizar las resinas dentales “bajo demanda” en la boca ha revolucionado la odontología moderna. En consecuencia, la unidad de fotopolimerización dental (UCL) se ha convertido en un equipo indispensable en casi todos los consultorios dentales ^46^.

### El peligro de la luz azul

Todas las lámparas de fotopolimerización dental pueden causar daño ocular por la luz azul que emiten. Este peligro de luz azul es mayor a 440 nm, que está dentro del rango de salida de las LCU dentales. Los profesionales dentales deben conocer y usar la protección adecuada contra este peligro porque tienen el deber de proteger tanto al paciente como a los empleados. La luz azul se transmite a través de los medios oculares y es absorbida por la retina ^47^.

### Consideraciones de temperatura

Es importante no aumentar arbitrariamente el tiempo de exposición para garantizar la fotopolimerización completa sin comprender los efectos térmicos potencialmente dañinos de la LCU. Contrariamente a las afirmaciones iniciales, las lámparas de curado de diodos emisores de luz (led) de alta potencia pueden producir aumentos significativos de temperatura. Cuando se comercializaron las luces led de primera generación, se anunciaban como luces “frías” que producían un aumento menor de la temperatura en la pulpa, en comparación con las luces de cuarzo-tungsteno-halógeno (QTH). Esto solo era cierto debido a la baja potencia de salida de las versiones iniciales de estas lámparas de fotopolimerización led de primera generación ^48^.

### Lámparas de fotopolimerización económicas y dispositivos médicos no regulados

La profesión dental ahora puede comprar equipos dentales, incluidas lámparas de fotopolimerización, directamente en línea y a bajo costo, sin utilizar un distribuidor aprobado. Muchas de estas lámparas de fotopolimerización “económicas” no están reguladas y, a menudo, utilizan guías de luz de pequeño diámetro (6-7 mm). Como se discutió en otra parte de este artículo, esto significa que, aunque a menudo brindan menos potencia, puede parecer que brindan los mismos valores de irradiación que las lámparas de fotopolimerización que usan puntas de mayor diámetro ^49^. Los perfiles del haz de luz de estas luces económicas también pueden ser inferiores en comparación con las luces más caras de los principales fabricantes, y es posible que la electrónica de estas unidades económicas no compense cualquier disminución en la salida de la batería. Por lo tanto, la salida de luz de algunas lámparas de fotopolimerización que funcionan con baterías no reguladas puede disminuir sin previo aviso durante la operación ^50^.

### Tiempo de exposición e intensidad de la luz

Cada tipo de lámpara ofrece una longitud de onda distinta y un espectro de activación característico. Las lámparas halógenas están entre las más comunes; algunas poseen cables y otras no, y manejan una longitud de 400 nm y 510 nm. Si la lámpara posee la potencia adecuada, es sometida a una prueba mediante un radiógrafo. Una desventaja en las lámparas de luz halógenas es que el bulbo, el reflector y el filtro pueden degradarse con el paso del tiempo, lo que interfiere con el poder en el rango de salida de la unidad de luz ^51^. El reflector de la lámpara puede perder sus propiedades reflexivas por la pérdida del material reflexivo o por la deposición de impurezas sobre la superficie. Los recubrimientos de los filtros pueden verse astillados, además de que los filtros por sí solos pueden sufrir de roturas. La pérdida de estas propiedades, típicamente, reduce el rango de salida de luz. Como fuente de luz más actual se utiliza un diodo emisor de luz azul (led) en las lámparas de fotopolimerización ^52^. Estas fuentes de luz se caracterizan por las siguientes ventajas: la fotopolimerización tiene lugar a temperatura ambiente, poseen una gran estabilidad mecánica, tienen una larga vida útil y el espectro de emisión es muy limitado. Cabe mencionar que tanto las lámparas inalámbricas como las de cables tienen una diferente intensidad de luz entre sus características que brindan una mayor exposición de fotopolimerización a mayor distancia, para poder operar a más piezas dentarias u otro tratamiento. Otra diferencia es el uso constante de la lámpara, ya que la inalámbrica tendrá un tiempo de uso limitante; luego se tendrá que cargar para seguir con el tratamiento o procedimientos próximos ^53^. La importancia del conocimiento de las ventajas y desventajas de una lámpara nos ayudará a saber si poseemos una en condiciones óptimas y así alargar su vida útil. Tanto las lámparas como los diferentes equipos requieren de un mantenimiento, pues de lo contrario habría una deficiencia en su uso ^54^. Entre estas deficiencias, uno de los aspectos es que, a una mayor cantidad de energía e intensidad, generará mucho calor. La temperatura no debe superar los 42 °C, pues un valor superior originará daño pulpar. La temperatura ideal de una lámpara led actual es de 38 °C ^55^.

Los fabricantes de resinas compuestas de relleno en bloque fotopolimerizables abogan por que sus RBC puedan fotopolimerizar adecuadamente en un solo incremento que tenga un grosor de hasta 5 mm, lo que ahorra tiempo y reduce potencialmente los defectos y la porosidad en la mayor parte de la restauración. El tiempo de exposición es un factor crítico que influye en la fotopolimerización de las restauraciones de resinas compuestas ^56^.

Hay muchas unidades de fotopolimerización disponibles para uso de los dentistas, que tienen diferentes diámetros de punta; brindan distintas potencias radiantes, salida radiante, espectros de emisión; y tienen diferentes perfiles de haz de luz ^57^. Incluso, si la punta de la lámpara cubre toda la restauración, la fotopolimerización de la resina puede verse comprometida negativamente si la luz no se emite uniformemente a través de la punta de luz, puesto que algunas regiones de la restauración pueden recibir luz insuficiente y algunas regiones pueden recibir valores de irradiancia muy altos ^58^. Los tiempos de exposición más largos deberían mejorar la fotopolimerización y, en última instancia, disminuir los efectos de cualquier falta de homogeneidad del haz de luz. 

### Profundidad de curado

La profundidad de curado es el nivel en el que el valor de dureza es igual al menos al 90% de la dureza de la parte superior de la resina. La parte más externa de la resina, más cercana a la lámpara, experimentará una fotopolimerización más completa porque contiene más fotoiniciadores y radicales libres que luego se convertirán en cadenas poliméricas ^59^, pero este concepto no debe confundirse con la fotopolimerización, que es el proceso químico mediante el cual los monómeros de una matriz de resina se juntan para producir moléculas pesadas llamadas polímeros, los cuales pueden ser lineales o tridimensionales ^60^. Por ejemplo, el 50%; de la energía luminosa que llega al material compuesto se pierde a solo 0,5 mm de profundidad, el 25%, a 1 mm; el 9%, a 2 mm; y el 3%, a 3 mm ^61^. Esta reducción de la resistencia da como resultado lo que comúnmente llamamos profundidad de curado.

Es así que la dureza de la resina en su porción más externa asegurará la fotopolimerización; sin embargo, no garantizará una correcta profundidad de curado ^62^.

Existen diversos factores que pueden ver afectada la profundidad de curado: El tipo de resina compuesta que se utiliza, los incrementos de resina (que no deben ser mayores a 2 mm de espesor), la distancia de la luz medida desde la punta de la lámpara hasta la superficie de la resina, el tiempo y la potencia de radiación de la lámpara ^63^.

Por esta razón, International Organization for Standardization (ISO), con el fin de asegurar la correcta profundidad de curado de las resinas compuestas durante las restauraciones, definió un método para definir el espesor máximo de los incrementos de resina compuesta, el cual se denomina oficialmente ISO 4049 ^64^. En esta norma internacional, se especifican los requisitos para los materiales restauradores a base de resina para el uso primario en restauraciones directas.

Por ende, la profundidad de curado es una medida de la eficiencia de la fotopolimerización, ya que el material no fotopolimerizado puede migrar al ambiente oral y causar reacciones alérgicas en algunos pacientes, así como estimular el crecimiento bacteriano alrededor de la restauración ^65^. Por tal motivo, la norma ISO 4049 exige que la profundidad de curado de estos materiales sea superior a 2 mm y la pérdida de material sin fotopolimerizar, inferior a 0,5 mm ^66^.

## DISCUSIÓN

Existe una relación inversa entre la distancia para fotopolimerizar el material y la radiación, lo que da como resultado una relación directa entre el tiempo y la distancia de fotopolimerización, y mantiene una exposición uniforme a la radiación ^67^. Asimismo, los valores de irradiación promedio para una ubicación determinada se ven afectados negativamente por una mayor distancia de fotopolimerización, a medida que la luz emitida diverge en una superficie más grande, puesto que la orientación de la punta de la unidad de fotopolimerización sobre los dientes en ciertas áreas afectará la radiación recibida ^68^.

Los efectos de la fotopolimerización de las lámparas led *polywave* y *monowave* presentan diferencias significativas entre la resistencia a la compresión de la resina fotopolimerizadas, con lámpara led de onda única y polivalente, donde los tipos de luz y lámpara influyen directamente en la resistencia a la compresión de las resinas compuestas ^69^. Además, la lámpara led *monowave* presenta mayor profundidad de curado que la led *polywave*, puesto que la primera emite un estrecho rango de longitud de onda de 420 a 480 nm, con un pico máximo de emisión de 468 nm, el cual es ideal para activar a la canforoquinona ^70^. Con respecto a las lámparas led *polywave* poseen longitudes de onda que pueden fotopolimerizar cualquier material, activando todos los fotoiniciadores agregados en las resinas compuestas actuales con un rango de 385 a 515 nm ^71^.

A medida que las resinas a base de canforquinona envejecen, se vuelven más amarillas, mientras que el PPD y la lucerina se vuelven más claras; sin embargo, los sistemas de canforquinona exhiben una mayor estabilidad de color que los fotoiniciadores de PPD y lucerina, aunque son más susceptibles a la fotoactivación, lo cual indica que el rango de longitud de onda corresponderá a una mayor eficiencia de curado ^72^. Entonces, el efecto de la combinación de canforquinona y luciferina sobre el color de la resina y las propiedades de fotopolimerización destaca que no existe una diferencia significativa en el grado de conversión entre resinas con canforquinona y luciferina a profundidades de curado de hasta 2 mm ^73^. Asimismo, concentraciones más altas de lucerina redujeron el amarillamiento de la restauración, mientras que concentraciones más bajas de canforquinona redujeron la absorción de luz en las capas más profundas ^74^. Respecto del PPD, es un fotoiniciador claro o translúcido con un espectro de absorción de 390 a 450 nm, con un pico máximo de 410 nm; la unión de este fotoiniciador con la canforquinona dará como resultado una mejor fotopolimerización de la resina y una disminución de amarillento residuales de las restauraciones ^75^. Shimokawa *et al*.^76^ menciona que fotopolimerizar en una restauración que tiene incrementos de 2 mm durante 20 segundos con una alta intensidad desde 1000 mW/cm^2^ sería adecuado, ya que las propiedades mecánicas de la restauración, como la microdureza, la integridad marginal y el estrés de contracción no se verían afectadas. Por tal motivo, el funcionamiento correcto y una intensidad adecuada de las lámparas de fotopolimerización led son fundamentales para la durabilidad y las propiedades físicas, mecánicas y ópticas satisfactorias de las resinas ^77^.

Los fabricantes de lámparas de fotopolimerización hacen que estos productos tengan una determinada potencia óptica o longitud de onda e intensidad, y las calibran con determinados tiempos de exposición y diferentes modos de fotopolimerización ^78^. No obstante, en algunos casos, no todos los materiales dentales son compatibles con estas lámparas, por lo que los odontólogos se encuentran con muchas preocupaciones al usarlos ^79^. Los profesionales, a menudo, están sobreexpuestos a la luz fotopolimerizada en el material, lo que puede afectar negativamente las propiedades mecánicas y físicas del material, ya que puede causar daño al tejido dental, daño endodóntico o iatrogenia, al aumentar la temperatura de la lámpara ^80^. Por consiguiente, cuando se vaya a utilizar el dispositivo de fotopolimerización, se recomienda utilizar un radiómetro para medir la intensidad de la luz de la lámpara led y determinar el tiempo de fotopolimerización adecuado ^81^.

Para alcanzar un tratamiento exitoso a base de resinas compuestas es importante una fotopolimerización de calidad que garantice un excelente desempeño clínico ^82^. Las lámparas de fotopolimerización deben cumplir dos requerimientos básicos: el primero, una longitud de onda capaz de activar el fotoiniciador de la resina en un rango de 400 nm a 515 nm; el segundo, emitir una irradiación que, multiplicada por el tiempo adecuado de exposición dentro de la longitud de onda antes mencionada, sea suficiente para fotopolimerizar los incrementos de resina ^83^. Sin embargo, si se utilizan lámparas que emitan valores iguales o mayores a 800 mW/cm^2^ por un tiempo de exposición de 40 segundos, tendremos una buena fotopolimerización, pero al mismo tiempo existiría el riesgo de causar daño tisular por exceder el valor de 24J/cm^2 (^^84^.

Por ende, es responsabilidad del profesional conocer la emisión de luz de su lámpara de fotopolimerización, de tal modo se establezca el tiempo de exposición obteniendo los valores correctos para una fotopolimerización exitosa, sin causar daños en tejidos vecinos a la restauración ^85^. Es importante polimerizar correctamente durante 20 segundos, pues de no ser así se produciría la desadaptación entre el adhesivo y la dentina, con la consecuente microfiltración en la restauración que podría generar una caries recurrente y el fracaso final de la restauración _^(86)^_ .

## CONCLUSIONES

El presente artículo arroja como conclusión que el uso adecuado de las diferentes lámparas led tendrá influencia en la eficiencia de la fotopolimerización de la resina. Asimismo, la fotopolimerización adecuada de las resinas compuestas está relacionada con la intensidad, la longitud de onda, el tiempo, la distancia, el espesor, la composición del material y el método de fotopolimerización. Las lámparas *monowave* emiten un estrecho rango de longitud de onda 420 a 480 nm, cuyo pico de espectro de emisión es próximo al pico de absorción de la canforoquinona, el cual es de 468 nm. Así, se concluye que las lámparas led con tecnología de onda única (*monowave*) producen una mayor profundidad de fotopolimerización que las de onda múltiple (*polywave*).

## Financiamiento:

No existen fuentes de financiación públicas o privadas en la realización del presente estudio.
